# Parents’ Perceptions of What Health Means for Adolescents with Depression—A Qualitative Study

**DOI:** 10.3390/children12091267

**Published:** 2025-09-21

**Authors:** Stina Persson, Emma Haglund, Rebecca Mortazavi, Håkan Jarbin, Ingrid Larsson

**Affiliations:** 1 School of Health and Welfare, Halmstad University, SE-30118 Halmstad, Sweden; stina@foretagshalsadirekt.se; 2 Department of Environmental and Biosciences, School of Business, Innovation and Sustainability, Halmstad University, SE-30118 Halmstad, Sweden; emma.haglund@hh.se; 3 Spenshult Research and Development Centre, SE-30274 Halmstad, Sweden; 4 Department of Clinical Sciences, Section of Rheumatology, Lund University, SE-22242 Lund, Sweden; 5 Child and Adolescent Psychiatric Clinic, Region Halland, SE-30185 Halmstad, Sweden; rebecca.mortazavi@regionhalland.se (R.M.); hakan.jarbin@regionhalland.se (H.J.); 6 Department of Clinical Sciences, Faculty of Medicine, Child and Adolescent Psychiatry, Lund University, SE-22184 Lund, Sweden; 7 Department of Health and Care, School of Health and Welfare, Halmstad University, SE-30118 Halmstad, Sweden

**Keywords:** adolescents, depression, health, parents, phenomenography

## Abstract

**Highlights:**

**What are the main findings?**

- Health for adolescents with depression was described in terms of managing everyday life, building confidence and trust, experiencing joy in life, and supporting well-being.
- These aspects reflect how health can be understood through daily routines, relationships, emotional experiences, and lifestyle habits.

**What is the implication of the main finding?**

- The findings may support the development of strategies that promote health among adolescents with depression by addressing how health is understood and supported in everyday life.
- A deeper understanding of how health is perceived in this context may inform health-promoting efforts in healthcare, schools, and family life.

**Abstract:**

Background: Adolescent depression is a growing public health concern. Health promotion for this group requires an understanding of what health means in the context of depression. Given that parents play a central role in adolescents’ everyday lives, their perspectives on health for adolescents are important. The aim of this study was to describe parents’ perceptions of what health means for adolescents with depression. Methods: This qualitative study employed a phenomenographic approach. In-depth interviews were conducted with 28 parents of adolescents with depression. The analysis resulted in four categories, each comprising two sub-categories, reflecting variations in how health was perceived. Results: Parents described health for adolescents with depression in terms of taking initiative in everyday life and creating a daily structure through routines (navigating daily life with depression); experiencing belonging in supportive relationships and understanding one’s own value (building trust in self and others); having a positive outlook while facing struggles and finding pleasure in daily activities (experiencing joy despite depression); and balancing body and mind and maintaining healthy habits (supporting well-being despite depression). Conclusions: The findings provide new insights into how parents understand health for adolescents with depression. These perceptions may inform the development of supportive and health-promoting strategies tailored to adolescents’ and their families’ everyday challenges.

## 1. Introduction

The rising prevalence of mental illness is a global public health concern, particularly among adolescents [1,2]. The increase is especially notable among girls and older adolescents [1,3]. International epidemiological studies estimate a point prevalence of 8% and lifetime prevalence of 19% for depression among 10–19-year-olds [1]. A U.S. study arrived at a one-year prevalence of depression in adolescents of 15.8% [4]. Depression is characterized by symptoms such as persistent low or irritable mood, lack of energy, and diminished motivation. To meet the diagnostic criteria, symptoms must persist for at least two weeks [5].

Adolescent depression impacts multiple areas of life and is associated with risk-taking behavior [6,7], academic difficulties [8,9], long-term labor market challenges [10], and cardiovascular disease [11]. It also increases risk of social isolation, which may be linked to impaired relationships with family and peers [12]. Suicide is a severe consequence of depression and the second leading cause of death among 10–24-year-olds [13], making it a major public health issue [14]. Adolescent depression affects not only the individual’s health and ability to manage daily life [5] but also those around them [15,16], particularly parents [12,17,18,19,20,21].

There are various definitions of health. According to Tengland (2007) [22], health comprises an individual’s basic abilities and subjective well-being, which mutually influence one another [22]. A healthy lifestyle is strongly associated with better self-rated health among adolescents [23]. Good self-rated health has a protective effect on mental well-being [18,19] and supports adolescents’ capacity to cope with emotional and situational challenges [24,25]. For adolescents in general, health is shaped by friendships and parental relationships [12,21,26], academic stress and performance [27,28], and personal goals [29]. A sense of belonging also contributes to adolescents’ health [29,30], and this has been emphasized by adolescents with depression [31]. These adolescents also highlight having an internal drive to engage in behaviors that support a healthy lifestyle, such as participating in life despite limitations [31]. Supportive family dynamics and parent–child relationships play an important role in the adolescents’ recovery process and the reduction in depressive symptoms [12,21]. Parents frequently initiate help-seeking for their child [17,18], they play a pivotal role in promoting adolescent health [15,16], and in shaping adolescents’ health perceptions [12,15,17,18,19,21]. The goal of depression treatment is to promote health [32]. A deeper understanding of adolescent health in the context of depression, explored from multiple perspectives, is therefore needed [19].

Previous research has mainly focused on general health-promoting aspects for healthy adolescents, such as positive peer relationships, family connections, and personal goals [23,27,29]. For adolescents with depression, previous research has identified a range of factors related to mental health. Risk factors were excessive screen time, substance use, and low appearance satisfaction [24], while strong social connections were protective [30]. Family satisfaction and resilience were important in mitigating symptoms of anxiety and depression [21]. One recent study explored how adolescents with depression understand health, describing it as the ability to manage daily routines, access social support, have control over depressive symptoms, and experience joy as a source of inner motivation [31]. Given the important role parents play in adolescents’ lives [18,21], their perspectives are essential. Parent perceptions can provide a more comprehensive understanding of how health is conceptualized and support the development of effective strategies for promoting adolescent health. However, studies exploring parents’ views on what health means for adolescents with depression are scarce.

Therefore, the aim of this study was to describe parents’ perceptions of what health means for adolescents with depression.

## 2. Materials and Methods

### 2.1. Study Design

This study employed a qualitative, inductive design with a phenomenographic approach to describe parents’ perceptions of what health means for adolescents with depression. Phenomenography aims to explore how a phenomenon is understood by individuals, focusing on qualitatively different ways of understanding rather than on the objective nature of the phenomenon itself. This involves adopting a second-order perspective, meaning that the study examines how people conceptualize the phenomenon in their lived world, rather than describing the phenomenon as it exists independently [33]. The study followed the COREQ criteria for reporting qualitative research (Supplementary Materials) [34].

### 2.2. Participants

Participants in this study were recruited through purposive sampling. Inclusion criteria were as follows: parents of adolescents with mild to moderate depression who were participating in an ongoing randomized controlled intervention trial (RCT) and a prior pilot RCT investigating the effects of moderate to vigorous-intensity group training on adolescents with depression [35]. Adolescents in those studies were randomized 1:1 to group exercise or leisure activities three times a week for 12 weeks; for details on inclusion and exclusion criteria, see the study protocol [35]. The recruitment of parents to the current qualitative study aimed to ensure variation in demographic characteristics among participants (Table 1). A total of 28 parents of adolescents from both the intervention (aerobic exercise) and control groups (leisure activities) were invited to participate, and all agreed to take part.

### 2.3. Data Collection

In-depth interviews were conducted with the parents between autumn 2021 and autumn 2023, after the adolescents had completed the intervention. An open-ended interview guide, which was pilot-tested and developed based on the aim of the study, was used. The main question was “From your point of view, what does health mean for your child?”, followed by prompts such as “How do you perceive your child’s health?”, “Can you describe what health means for your child?”, and “Describe what influences your child’s health”.

The interviews were conducted individually via telephone or digitally via Zoom by the last author (n = 20), an experienced qualitative researcher and professor, and some by a psychologist (n = 8) under the supervision of the last author. This ensured both consistency in the interview approach and professional competence in the data collection. The interviewers had no prior relationship with the participants. The research team continuously reflected on their own professional backgrounds and assumptions related to adolescent health. These reflections were discussed throughout the project to maintain awareness of potential preconceptions and their influence on data collection and interpretation. Interview duration ranged from 35 to 85 min, with a total interview time of 18 h and 59 min. All interviews were carried out and analyzed in Swedish, audio-recorded, transcribed verbatim, and anonymized prior to analysis. A professional, authorized translator subsequently translated the analysis into English to ensure accuracy and preserve the semantic nuances of the quoted extracts.

### 2.4. Data Analysis

The transcribed material formed the basis of the analysis, which followed the first six steps of phenomenographic analysis as described by Larsson and Holmström (2007) [36]. The analysis was inductive and conducted manually in Microsoft Word. Before formal analysis began, the research team shared and discussed their respective perspectives and professional experiences in relation to the study context. These initial reflections helped raise awareness of potential influence on interpretation and contributed to an open and reflective approach during the analysis process.

Step one involved familiarization with the material by thoroughly reviewing the transcribed data. In step two, the full text was re-read to identify and extract 210 statements, describing perceptions that reflected parents’ ways of understanding the meaning of health for adolescents with depression. In step three, the material was condensed and preliminary coded while retaining the core meaning. Each condensed statement was assigned a code representing a preliminary description of each parent’s perception of health. After discussion in the research group, a total of 43 codes were defined. These codes were compiled into a working code list that was adjusted during the analysis as new nuances emerged. Coding decisions were continuously discussed and refined in collaboration between the authors. Throughout the analysis, no new codes or categories emerged after approximately the 20th interview, indicating that a sufficient range of perceptions had been captured. To ensure robustness, 28 interviews were conducted, allowing for a comprehensive examination of the phenomenon. In step four, the codes were grouped based on similarities and differences, resulting in eight sub-categories reflecting different understandings of the phenomenon of health. Examples of the coding process include how the codes “Take own initiative”, “Handle things”, and “Be motivated to do things” were grouped to form the sub-category “Taking initiative in everyday life”. The analysis was iterative, moving back and forth between steps through ongoing discussions among the co-authors. These sub-categories were then organized into four categories. The categories were iteratively compared to ensure that each represented a distinct way of understanding at a comparable level of abstraction. Differences in interpretation were resolved through discussion, and coding decisions were guided by comparisons with previous codes and categories. While no predefined decision rules were applied, consensus was reached through repeated group discussions and by returning to the data. Final agreement on the categories was reached through a process of negotiated consensus among the researchers to enhance dependability (Table 2).

Step five involved identifying dominant and non-dominant ways of understanding the phenomenon by interpreting which category was most prominent (dominant) in each interview, rather than quantifying the categories. Non-dominant perceptions were also explored across all interviews to identify additional patterns. Step six involved structuring the outcome space, that is, examining the underlying structure and relationships between the categories. No hierarchical relationship was found. The outcome space represents the parents’ perceptions of the meaning of health for adolescents with depression and constitutes the study’s findings.

The first author conducted the analysis to ensure consistent application of codes, with the second and last authors as co-assessors. Differences were discussed throughout, and all authors engaged in reflexive discussions of potential preconceptions until consensus was reached. Coding decisions, analytical memos, and meeting notes were documented to strengthen dependability and confirmability, and interpretations were anchored in verbatim data. Notes from analytical discussions and coding sessions were saved to support transparency and traceability in the analysis. Although not structured as a formal audit trail, these materials contributed to the overall rigor of the study. The authors have extensive experience in qualitative research, in the field of health and lifestyle, and in child and adolescent psychiatry. The first author holds a Master’s degree in health and lifestyle; the second, a PhD, is a senior lecturer; and the last author, a PhD, is a professor in health and lifestyle. The fourth author is the project leader of the RCT. The third and fourth authors are clinically active psychiatrists, the former being a PhD student and the latter being a PhD. They aimed to prevent having clinical contact with the families included in the study, although such contact may have occurred in exceptional cases, where it could not be avoided.

### 2.5. Ethical Considerations

The study was approved by the Swedish Ethical Review Authority (nos. 2020–03364 pilot study; 2021-04029, supplementary application for adding parental interviews to pilot study; 2021-05307-01, multicenter RCT; 2024-04975-02, amendment for changes in multicenter RCT) and adhered to both the principles outlined in the Declaration of Helsinki [37] and the ethical guidelines of the Swedish Research Council [38]. Written informed consent was obtained from all participants, and they received verbal and written information about the study’s purpose, procedures, and their rights, including the right to withdraw at any time [35]. Confidentiality was ensured by restricting access to study materials to authorized research team members only. All data were de-identified and securely stored in accordance with the General Data Protection Regulation [39].

## 3. Results

This study identified four qualitatively distinct ways in which parents understood what health means for adolescents with depression. In their accounts, health was related to various aspects of everyday life. The following four categories emerged: 1. navigating daily life with depression, consisting of taking initiative in everyday life and creating daily structure through routines, 2. building trust in self and others, consisting of experiencing belonging in supportive relationships and understanding one’s own value, 3. experiencing joy despite depression, consisting of having a positive outlook while facing struggles and finding pleasure in daily activities, and 4. supporting well-being despite depression, consisting of balancing body and mind and maintaining healthy habits. Each category represents a distinct way of understanding health, with sub-categories that capture variations in meaning. Together, they constitute the outcome space of the study.

### 3.1. Navigating Daily Life with Depression

Health was understood as involving various ways of navigating daily life with depression. This included taking initiative in everyday life, where adolescents were described as initiating activities and making independent choices, and creating a daily structure through routines that supported stability in day-to-day life.

#### 3.1.1. Taking Initiative in Everyday Life

Parents described the meaning of health for adolescents with depression as involving the ability to take initiative in everyday life. This included making independent decisions that contributed to managing daily routines. Reaching out to others, especially peers in similar situations, was perceived by parents as an expression of initiative and as supporting social connection.

*I can tell [that they are doing well] when they reach out. They’re happy and want to do things and so on.*

(Mother of daughter, ID 5)

Taking initiative in everyday life was described as connected to adolescents’ motivation, driven by goals, interests, a wish to feel better, or a sense of responsibility. Parents highlighted leaving the house and engaging in new environments as important for strengthening routines and managing depressive symptoms.

*It’s positive that he has participated in [the leisure group activity]. He used to go fishing before, but he got tired of that. Now, he has had this [activity] to focus on, so that’s been good. He has been able to get out somewhere; he hasn’t just been sitting at the computer at home.*

(Mother of son, ID 4)

Taking initiative also included attending school. Parents emphasized that this was less about academic achievement and more about finding the energy and courage to go. Adolescents’ willingness to take responsibility and complete tasks often led to increased confidence and pride. According to the parents, this pride contributed to adolescents’ sense of capability and health. Trying one new activity could inspire further attempts, reinforcing a sense of agency.

*As long as you feel that you can manage it, I think. Or you increase gradually and then you realize that you can handle more and more. Then it becomes a positive effect when you see that you can do more than you thought.*

(Mother of daughter, ID 16)

Taking initiative in everyday life was thus described as one strategy that adolescents used to manage everyday life and navigate their health in the context of living with depression.

#### 3.1.2. Creating Daily Structure Through Routines

Creating a daily structure was also described by parents as a meaningful strategy for adolescents with depression. Routines around school, leisure, meals, and rest helped establish predictability and reduce uncertainty.

*[Routines are important]. He doesn’t like it when it comes as a surprise. He wants to know in advance. It is easier for him that way.*

(Mother of son, ID 22)

Structure was perceived as helping adolescents organize their time and maintain a sense of order. Having a predetermined plan for the day was mentioned as helpful in giving the day meaning and allowing adolescents to prepare for what was to come. This planning was said to increase the likelihood that activities would be carried out. Parents described daily routines as contributing to emotional security and supporting adolescents in coping with fluctuations in energy and mood.

*I’d say they just make up their mind to do it. That process is pretty difficult for them./…/So it’s really the mental preparation that’s important.*

(Mother of daughter, ID 20)

A structured balance between activity and recovery was considered beneficial. Adjusting expectations and focusing on participation rather than performance were described as ways to reduce pressure and support mental health. Focusing on something external, such as a hobby or time with others, also seemed to help adolescents redirect attention away from illness-related thoughts.

*To be able to handle everyday life, to not just go to school every other day.*

(Mother of daughter, ID 15)

According to the parents, daily structure provided a foundation that enabled adolescents to maintain functioning and supported them in developing routines that promoted health in everyday life.

### 3.2. Building Trust in Self and Others

Health for adolescents with depression was also described by their parents in terms of building trust in self and others. These aspects were understood to develop gradually in relation to others. Health was expressed through experiencing belonging in supportive relationships and understanding one’s own value, both of which contributed to an increased sense of stability and self-assurance in everyday life.

#### 3.2.1. Experiencing Belonging in Supportive Relationships

Parents described health in adolescents with depression as linked to a growing sense of belonging, which in turn contributed to building confidence and trust. Belonging was understood as developing supportive relationships with others, particularly with family, siblings, and peers with similar experiences or shared interests, but also with pets. These relationships were seen as reducing feelings of isolation and increasing understanding.

*For her, health means having animals around her, her family around her, and above all, her siblings; those are probably the two most important aspects, and also some friends, but especially her siblings.*

(Father of daughter, ID 24)

Conversations with others in similar situations were described as providing opportunities to exchange experiences, find hope, and recognize different ways of coping. Group settings with a safe and accepting atmosphere were perceived by parents as enabling adolescents to take part more fully. A warm, non-judgmental approach from others contributed to a sense of trust and supported the adolescents in being themselves.

*I think there’s a different level of understanding [among other children in the same situation]. You can be more yourself. There’s more acceptance when someone is struggling or doesn’t fit the mold. There’s no expectation to conform.*

(Mother of daughter, ID 21)

Parents also noted that social participation often led to a more positive mood and increased motivation to reach out. Even when adolescents were initially hesitant, engaging in shared activities with others was described as encouraging further involvement and strengthening social connections.

*[Health] is about being part of a context and being somewhat active in it. It’s not just the absence of illness that defines health.*

(Father of daughter, ID 23)

According to the parents, developing a sense of belonging appeared to be part of how adolescents with depression regained confidence in themselves and trust in others, both seen as aspects of their health.

#### 3.2.2. Understanding One’s Own Value

Understanding their own value was described by parents as another aspect of how adolescents built confidence and trust. When adolescents dared to express themselves, approach others, or participate in new situations, parents considered these acts to be signs of personal growth and a developing sense of self.

*Yes, it’s about being able to participate in social settings again, to speak up, even in front of people they don’t know that well, to express their opinions. Their social skills have improved, you could say.*

(Mother of daughter, ID 3)

Feeling proud after overcoming challenges or succeeding in everyday tasks was understood as contributing to a more positive self-image. Parents observed that this sense of pride could help reduce self-criticism and support emotional stability. These moments were seen as steps toward greater self-acceptance and autonomy. Situations where adolescents felt seen, listened to, and invited to contribute were described by parents as supporting their understanding of their own value.

*[Health is] to be seen and respected. For her, it’s to be listened to, based on her own perceptions and thoughts, and so on./…/She would need people who are very open-minded and don’t judge different perspectives, but can understand and appreciate different viewpoints on things. Then she likely experiences being seen and respected.*

(Father of daughter, ID 25)

When their input was welcomed in peer groups or everyday contexts, adolescents seemed to gain confidence and trust in themselves and others. Feeling accepted by others, especially in peer groups, was described by parents as helping adolescents recognize their value and capabilities. Being acknowledged, invited to contribute, and encouraged in their efforts supported a growing trust in their abilities and relationships with others.

*I think they’ve become stronger in themselves [that’s why they dare]. Because they’ve had so much support around them, lifting them up, so to speak.*

(Mother of daughter, ID 10)

According to the parents, this developing understanding of personal value supported adolescents with depression in navigating everyday challenges and contributed to a broader process of building confidence and trust.

### 3.3. Experiencing Joy Despite Depression

Another way of understanding health for adolescents with depression was through the experience of joy in everyday life despite depression. This understanding included emotional and motivational aspects that were expressed in a more positive outlook and in finding pleasure in daily activities.

#### 3.3.1. Having a Positive Outlook on Life While Facing Struggles

Parents described health as involving the adolescents’ ability to experience joy and approach life with a positive outlook. This included emotions such as optimism, playfulness, and light-heartedness. Parents observed that such moods often arose when adolescents were socially engaged or took part in meaningful activities. On these occasions, adolescents were perceived as more present in everyday family life.

*[Health is] how much they socialize and talk./…/On the days when things are better, they’re much more talkative. Like at home, they’re much more present with the rest of us, not just in their room.*

(Mother of daughter, ID 9)

Having a positive outlook was also expressed through the ability to imagine a future. Adolescents who talked about their hopes or dreams, whether related to education, friendships, or future relationships, were described as demonstrating regained motivation. Encounters with others in similar situations were seen as contributing to this outlook by offering role models and alternative perspectives.

*I think they’ve spent a lot of time thinking about what they want from life. They want to keep going. They want to continue. They want to go to school and do what they enjoy.*

(Mother of son, ID 1)

Experiencing joy in life, as understood by the parents, involved more than momentary happiness. It was described as part of a broader orientation toward life that reflected emotional presence, forward-looking thinking, and the re-emergence of motivation in adolescents with depression.

*Health is having a vision of one’s future where he sees that he can achieve it.*

(Mother of son, ID 7)

#### 3.3.2. Finding Pleasure in Daily Activities

Joy in everyday life was also described as arising when adolescents with depression found pleasure in daily activities that felt enjoyable or meaningful. According to the parents, such activities included spending time with friends, listening to music, engaging in creative pursuits, being in nature, or caring for animals. These moments were perceived as supporting emotional balance and offering relief from distressing thoughts.

*Yes, [health is] when they get to do things they enjoy, really.*

(Father of son, ID 4)

Participating in leisure activities was seen as helping adolescents shift their attention away from illness and toward more positive experiences. Parents emphasized that this shift often led to more varied conversations at home, where the focus moved beyond symptoms or challenges. Involvement in activities also appeared to increase the adolescents’ sense of vitality and presence.

*I think they liked [going to the movies], even if we had to push them a bit, but they went, and then they had something to talk about. It’s different when they’ve experienced something themselves compared to just hearing about it. It creates a different level of engagement.*

(Mother of daughter, ID 6)

According to the parents, finding pleasure in daily activities was part of how adolescents with depression reconnected with their surroundings and experienced moments of pleasure and connection. These experiences were described as contributing to a gradual return of emotional engagement and joy in life.

### 3.4. Supporting Well-Being Despite Depression

Health was further understood as being supported by conditions that enabled physical and emotional balance. This was expressed through balancing body and mind, where emotional regulation and energy levels were central, and maintaining healthy habits, which involved sleep, nutrition, and physical activity routines.

#### 3.4.1. Balancing Body and Mind

Supporting well-being was described by parents as involving a state of balance between emotional and physical functioning. Adolescents with depression were perceived as experiencing greater calm and stability when they had access to meaningful activities or when their everyday routines supported predictability.

*[Health is] having the energy to get up in the morning.*

(Mother of daughter, ID 18)

Emotional regulation included the ability to reflect and respond constructively to challenges. Parents observed that a calmer emotional state appeared to reduce anxiety and stress and contributed to the adolescents’ ability to manage symptoms of depression. When they were able to assess situations and avoid emotional escalation, this was interpreted as a sign of improved well-being. The capacity to remain present and composed was described as developing over time, often in connection with gaining insight into their own feelings and reactions.

*He has better balance in life because somehow he now knows himself and his emotions better, and he doesn’t have to go to those extremes. Overall, he can now assess the situation, reflect on it, and respond in a way that doesn’t make things worse.*

(Mother of son, ID 7)

In addition to emotional aspects, parents also described physical well-being as part of supporting balance. Having energy to participate in daily life, feeling physically fit, and maintaining a healthy body weight were seen as contributing factors. A balanced rhythm between rest and activity was perceived as essential for managing everyday life with depression.

*[Having the strength to participate] can be anything; life, activities, school, things that he himself has chosen and wants to do that make him feel good. That he has the energy to take part in it. That he can manage it. That is simply functioning. That is health. You feel better when you can do the things you want to do.*

(Mother of son, ID 14)

According to the parents, when adolescents experienced both emotional stability and physical energy, it supported a broader sense of well-being.

#### 3.4.2. Maintaining Healthy Habits

Maintaining healthy habits was described by parents as one way in which health can be understood for adolescents with depression. These included habits related to physical activity, diet, and sleep, which were perceived as interrelated.

*[Health comprises] regular sleep, exercise, and food. If you can keep those at a good level, I think you get everything else automatically, or at least most of it.*

(Father of daughter, ID 19)

Physical activity was described as influencing mood, energy, and self-confidence. Some parents noted that exercise, whether performed individually or in groups, contributed to increased activity and participation. Physical sensations experienced during exercise were also mentioned as a possible way to shift focus from emotional discomfort. Dietary habits were described as part of the daily rhythm and connected to energy levels and the ability to participate in daily activities. Eating regularly and maintaining a varied diet were highlighted as contributing to a more structured day.

*[Health] is about energy intake and diet, eating properly.*

(Father of daughter, ID 23)

Sleep was also described as a relevant aspect of health. Parents perceived that improved sleep supported adolescents’ ability to manage daily routines and contributed to a more even mood.

*And when he gets into a better sleep rhythm, falling asleep in the evening instead of in the middle of the night, everything gets easier. It’s easier to get to school./…/Establishing a routine, getting tired at night, eating and sleeping better, all of that has been positive./…/It has given him some structure.*

(Mother of son, ID 22)

Maintaining healthy habits was thus described as a part of how health can be understood for adolescents with depression, where habits around physical activity, diet, and sleep formed elements of a structure that supported well-being.

### 3.5. Outcome Space

**Figure 1:**
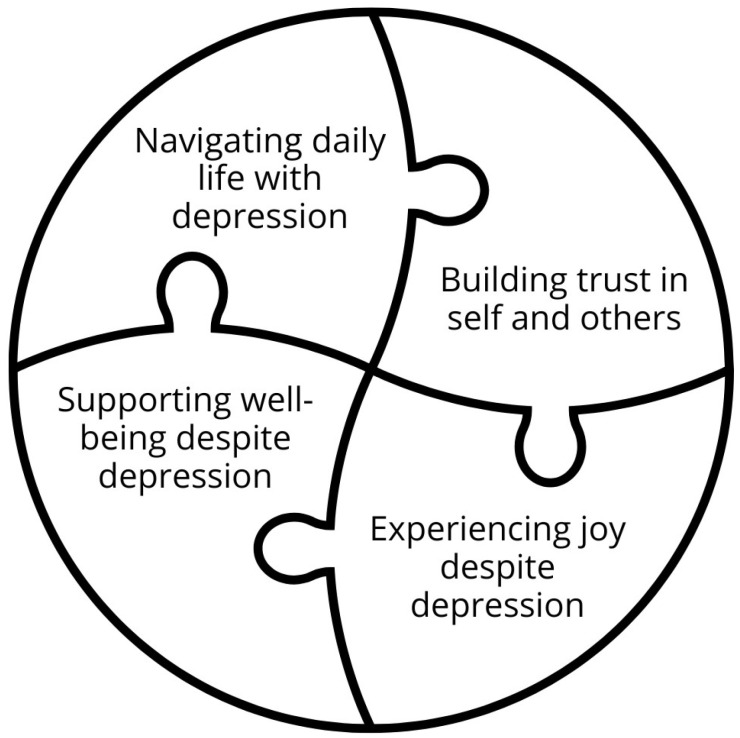
Overview of the outcome space illustrates how the four categories are interrelated and form a cohesive understanding of what health means for adolescents with depression, based on parents’ perceptions. The categories are related to one another in a non-hierarchical way, reflecting their equal importance in the outcome space.

The outcome space represents the result of the phenomenographic study and illustrates how the four categories, navigating daily life with depression, building trust in self and others, experiencing joy, and supporting well-being despite depression, are interrelated and together shape parents’ understanding of what health entails for adolescents with depression (Figure 1). The outcome space is based on the collective variation in understanding and does not reflect individual differences [36]. Each category represents a distinct way in which health is experienced and supported. The categories are interrelated parts of a whole, influencing and reinforcing one another in various ways. Their relationships are non-hierarchical, as each can both contribute to and be strengthened by the others.

Navigating daily life with depression was described as giving adolescents direction and predictability, which in turn supported their ability to trust themselves and others, experience joy, and feel well. At the same time, building trust in oneself and others was seen as enabling adolescents to take initiative and find pleasure in daily activities. Experiences of joy despite depression were described as having a positive outlook on life while facing struggles and finding pleasure in daily activities, which supported daily functioning and contributed to emotional balance. Likewise, supporting well-being, through healthy routines and emotional stability, was perceived as both a condition for and a result of navigating daily life, building trust, and experiencing joy despite depression.

In this way, the outcome space forms a dynamic whole, where the categories are closely connected and mutually reinforcing. The non-hierarchical puzzle illustration was chosen to reflect their equal importance and interwoven nature, where any one category can contribute to, or be strengthened by, the others.

## 4. Discussion

This phenomenographic study explored parents’ perceptions of what health entails for adolescents with depression. The findings revealed four interrelated ways in which health was understood: navigating daily life with depression, building trust in self and others, experiencing joy, and supporting well-being despite depression. These dimensions reflect how health is viewed as emerging through the interplay between practical strategies, emotional security, and psychosocial resources.

Navigating daily life with depression was described as taking initiative, creating daily structure through routines, engaging socially, and acting with responsibility. This finding aligns partially with previous research on adolescents’ own perceptions of health, where an internal drive was described as contributing to health through engagement in valued activities [31]. While adolescents emphasized finding purpose [31], parents stressed conscientiousness and age-related expectations, which reflects their more external observations. Previous research has also shown that responsibility and academic pressure may contribute to mental health concerns, particularly among girls [20]. This raises questions about how parents’ interpretations of proactivity relate to adolescents’ experiences of stress. Both groups stated that motivation and initiative were linked to engagement outside the home. This supports the idea that navigating daily life is both a prerequisite for, and a manifestation of, health [22].

Daily structure, such as predictable routines around sleep, meals, school, and social activities, was seen as essential to managing everyday life. These findings align with research that highlights routines as important for mental health and emotional regulation among children and adolescents [40]. In the current study, routines were not only seen as supportive of functioning but also provided security and predictability, features parents perceived as necessary for health in daily life. The parents’ descriptions suggested that structure played both a stabilizing and enabling role in adolescents’ lives, providing a framework within which recovery and participation became possible. Research further indicates that such routines often originate in family contexts, suggesting that parental involvement may influence adolescents’ health, not only indirectly but also through the co-creation of daily habits [40].

Another aspect of health was adolescents’ ability to build trust, both in themselves and with others. This included a sense of belonging and understanding of one’s own worth. A sense of belonging has been identified as a protective factor for mental health in previous studies [29,30,31] and has been described by parents as enabling social engagement and mitigating withdrawal and isolation [41]. Trust was linked to emotional safety, particularly in peer relationships, consistent with research on psychological safety and fear of rejection [42], as adolescents with depression often struggle with trust and fear of rejection in social situations [43]. Using group settings in treatment for adolescent depression may be a way to meet people with similar experiences and enable new social contacts outside the sessions. While adolescents themselves also highlight the importance of social inclusion, they emphasize material conditions and autonomy [29], elements not mentioned by the parents in the present study. This may indicate that parents focus more on relational and emotional aspects of health, while adolescents consider a broader range of health-related factors.

Self-worth was described as enabling healthier choices, initiative, and resilience, abilities often diminished by depression. This is consistent with research linking self-esteem in early adolescence to psychological well-being [44] and supportive networks [45], which were also identified by parents in the present study as crucial to health. Patterns of depression and self-esteem that differ between girls and boys may influence self-worth, as girls are more likely to experience depression during adolescence [1] and to report lower self-esteem than boys [44]. This highlights the need for social support tailored to young girls [43].

Experiencing joy in life despite depression was understood as a component of health, including vitality and a hopeful outlook on the future. This form of joy appeared to be closely linked to engagement in meaningful or enjoyable activities and to positive affirmation from others, echoing findings that group activities foster belonging and joy among adolescents with depression [31,46]. Encouragement and recognition were described as helping them maintain a more positive outlook, which is consistent with adolescents’ accounts of how external support fosters emotional resilience and vitality [31]. While optimism and a positive mindset have been associated with improved mental and physical health and good academic performance [47], parents did not explicitly link this to academic achievement, unlike findings in studies involving adolescents themselves [47,48].

Parents also described activities in nature or with animals as fostering joy, which is supported by studies linking animal contact to reduced loneliness [49,50,51] and nature to improved mental health [50]. The inclusion of such elements in parents’ perceptions of health indicates a recognition of alternative pathways to emotional recovery that extend beyond conventional clinical or school-based settings. However, research suggests that adolescents are spending increasingly less time outdoors [52], which could imply a tension between parents’ ideals and the everyday realities that adolescents encounter.

Supporting well-being was described as striving for physical and mental balance, achieved through motivation, emotional stability, and a healthy lifestyle. Well-being was seen as both an outcome of, and a precondition for, other aspects of health, which reflects a holistic understanding. Motivation was described as being interwoven with emotional and physical energy, enabling adolescents to sustain health-promoting behaviors over time. This aligns with research suggesting that purpose in life and future orientation may be protective factors against depressive symptoms in adolescence and young adulthood [53]. Parents’ descriptions connect motivation with vitality, highlighting well-being as both a mental and physical state. A healthy lifestyle, including sleep, physical activity, and diet, was considered central, consistent with evidence on lifestyle behaviors and adolescent mental health. This suggests that well-being, as conceptualized by parents, is not a fixed state but a dynamic experience shaped by how adolescents respond to and engage with everyday conditions [23,24,46,54]. However, aspects such as screen time or use of substances, which are commonly discussed in relation to adolescent health [23,24,55], were not brought up by parents in this study. This absence may reflect the study’s health-oriented focus, or it may suggest that parents conceptualize health more through the presence of protective factors than the absence of risk behaviors. Physical activity was described as beneficial, not only for physical health but also for improving sleep and mood, findings supported by earlier studies on adolescents with depression [46,56].

Together, the four categories reflect a dynamic view of health, shaped by daily strategies, emotional resources, and lifestyle behaviors. The mutual reinforcement suggested between the categories reflects a potentially multifaceted understanding of how everyday life management and joy in life may contribute to well-being, confidence, and motivation. Rather than being separate elements, these categories form an integrated system of meaning in how parents understand adolescent health. This is in line with Tengland’s (2007) definition of health as the interplay between an individual’s basic abilities and subjective well-being [22].

This study highlights differences between parents’ perceptions, as identified in the present analysis, and adolescents’ perceptions reported in a previous study [31]. Parents tend to focus more on external structure and relational aspects, while adolescents emphasize autonomy and internal meaning-making [31]. These differences underscore the need for support strategies that address both viewpoints. Moreover, the findings reveal inherent tensions, such as balancing supportive daily routines with adolescents’ growing desire for autonomy, which present challenges for families and practitioners. Framing these results within a family-centered care approach, which recognizes the family as a key resource and unit of care, aligns well with the multidimensional and interactive understanding of health demonstrated in the present study. This perspective reinforces the importance of involving families in interventions and tailoring support to the unique needs and dynamics of each adolescent’s context [57].

In advancing knowledge within this qualitative framework, this study contributes insights by articulating diverse parental conceptualizations of health that extend beyond individual adolescent experiences to encompass family systems and everyday life strategies. These findings provide a valuable foundation for developing holistic, family-oriented support that can better address the complexity of adolescent depression.

### 4.1. Practical Implications

The findings provide insights into health-promoting practices to support adolescents with depression, their families, and practitioners. Concerning navigating daily life with depression, the parents pointed out the importance of structured daily routines. Tools such as templates for scheduling meals, sleep, school, and leisure activities might serve as valuable aids in creating predictability and stability, as well as contribute to adolescents’ ability to cope with day-to-day challenges. Regarding building trust in self and others, the data suggests that fostering social belonging and providing opportunities for peer interaction may play a significant role. Facilitating access to support groups or organized activities within clinical or community settings could help adolescents feel connected to others facing similar experiences, thus nurturing emotional security and motivation. In terms of experiencing joy in life, encouraging involvement in activities that adolescents find enjoyable or meaningful may help enhance positive effects and resilience. Providing examples or suggestions of such activities for families might assist in integrating moments of pleasure and engagement into daily life. Well-being could be supported through regular physical activity, which may help improve energy, mood, and emotional regulation for some adolescents. Encouraging adolescents to engage in suitable exercise and incorporating guidance on maintaining healthy nutrition and sleep routines can empower families to foster holistic well-being. These practical insights may inform the development of family-centered, accessible support strategies tailored to the needs of adolescents living with depression.

### 4.2. Strengths and Limitations

The trustworthiness of the study was evaluated based on four established criteria: credibility, dependability, confirmability, and transferability [58,59]. Credibility, which concerns the truth value of the findings and the confidence that they reflect participants’ perceptions [59], was supported by a purposive sampling strategy. This approach enabled the inclusion of participants likely to provide rich and varied data relevant to the phenomenon under study. A total of 28 parents participated, which aligns with recommendations for achieving sufficient variation in a phenomenographic study [36]. Credibility may have been influenced by the fact that participants were recruited from an intervention study. This recruitment could have affected the parents’ perceptions, depending on their child’s allocation to the intervention or control group. This was addressed through careful sampling and a focus on capturing a broad range of experiences. The fact that the recruitment was from an RCT study based on exercise for depression may also have had an impact on the study population through selection bias, as families interested in a study on exercise may have different views on health than those not interested. Parents’ views on exercise might also have influenced their answers during the interviews and thus had an impact on the resulting themes. Further, the adolescents’ group allocation may have altered the parents’ experiences and thus their views on health. The adolescents in this project were allocated equally to either the exercise (n = 13) or the leisure group (n = 15), and thus experiences from both groups are addressed. In addition, a detailed description of the study context and participant characteristics, along with the use of in-depth interviews, further supported the credibility of the findings. Dependability relates to the consistency and stability of the research process over time [58]. Although two different individuals conducted the interviews independently, they both followed the same semi-structured interview guide and received consistent instructions to ensure coherence in data collection. Moreover, the separation of roles between data collection and analysis contributed to transparency and supported dependability. The analytical process was also documented in detail, so the steps taken during interpretation can be clearly traced. Confirmability refers to the extent to which the findings are shaped by the participants rather than by researcher bias or personal perspectives [59]. This was supported through a transparent account of the methodological approach and the inclusion of quotations from participants, which helped to anchor the interpretations in the data. Using participants’ own expressions strengthens the connection between the findings and the data, enhancing confirmability. In addition, the research team represented a range of professional backgrounds, including clinical psychology, child and adolescent psychiatry, and qualitative health research. These diverse perspectives contributed to a rich interpretative process, and the team made conscious efforts to reflect on how their respective positions and experiences could influence the analysis. Transferability concerns the extent to which the findings may be applicable in other contexts or settings [58,59]. This was supported through the provision of thick descriptions, including detailed accounts of the study context and participant characteristics. One limitation is that the sample primarily consisted of mothers and mainly parents of girls, which makes the findings more applicable to similar groups. However, depression is more prevalent among girls, and the sample in this study reflects this distribution [4]. A previous study on depression screening indicated that parental ratings were more informative for girls with depression than for boys [60]. The uneven representation of mothers and fathers among the interviewees may have influenced the results in the present study. The predominance of mothers and daughters may have shaped the interpretations through gendered parenting roles or communication styles. With only five fathers, mostly discussing daughters, the study may not fully capture paternal perspectives or experiences of parenting sons. These aspects should be considered when assessing the relevance of the findings.

Furthermore, as participants were recruited from an RCT context, that study setting may have influenced how they articulated their accounts, for instance, by focusing on aspects related to the intervention. However, parents primarily described what health means for their adolescents in general terms rather than in relation to the intervention, which suggests that the influence of the recruitment context was limited. Importantly, the quality of the adolescent–parent relationship is likely to be a significant factor. Previous research has shown that, for adolescents with internalized problems such as depression, the parent–child relationship correlates with the adolescents’ well-being [61]. In any case, the quality of the relationship is difficult to assess and not evaluated in this study. Including participants from both urban and rural areas across different regions of Sweden added variation in background and experience, which may support the transferability of the findings within similar Swedish contexts. Contextual details have been provided to facilitate readers’ assessment of transferability. Nevertheless, the applicability of the findings to other countries and healthcare systems should be considered with caution.

## 5. Conclusions

Parents described health as involving the ability to navigate daily life with depression, build confidence and trust, experience joy in life, and support a sense of well-being. These aspects were mutually supportive in enabling adolescents with depression to participate in daily life and regain a sense of direction, connection, and emotional balance.

By making visible how health is understood from different perspectives, this study adds parents’ perceptions to those of adolescents that have been previously explored. Together, these perspectives provide knowledge that may inform health-promoting and supportive measures tailored to adolescents’ everyday challenges. Further research is needed to explore how these findings can be translated into practical approaches in healthcare, school, and family settings, to examine their relevance beyond a Swedish context.

## Figures and Tables

**Table 1:** Demographic description of the participants included in the study (n = 28).

Variables	Parents (n = 28)
**Gender**	
Women/men (n)	23/5
**Age;** median (min–max), years	48 (32–57)
**Household type**	
Single parent/Cohabiting parent (n)	9/19
**Level of education**	
Primary school/upper secondary school/university (n)	2/11/15
**Employment**	
Full-time/part-time/on sick leave (n)	21/5/2
**Place of residence**	
City/countryside (n)	17/11
**Gender of the adolescents**	
Girls/boys (n)	23/5
**Age of the adolescents;** median (min–max), years	16 (14–18)
**Intervention group of adolescents**	
Exercise/Leisure activities (n)	13/15

**Table 2:** Examples from the data analysis for each category with corresponding perception, code, and sub-category.

Statements	Codes	Sub-Categories	Categories
Mother of daughter, ID 5: “Well, then she can take her own initiative and want to do things. I believe that is the key to wanting to do things, because that is when the person feels well.”	Take own initiative	Taking initiative in everyday life	Navigating daily life with depression
Mother of son, ID 7: “I think that routines, fixed routines, fixed studies provide a space to feel secure, to act from.”	Have regular habits	Creating a daily structure through routines	Navigating daily life with depression
Mother of son, ID 22: “He is social./…/. So, he can be with his friends./…/. But he needs that stimulation, to socialize with peers and so on.”	Participate in social contexts	Experiencing belonging in supportive relationships	Building trust in self and others
Mother of son, ID 1: “My child is still proud of themselves [for having turned their mental health around]. That they have reached the point of saying ‘I will,’ ‘I want.’”	Be proud of oneself	Understanding one’s own value	Building trust in self and others
Mother of daughter, ID 9: “She has applied to [school] and is thinking about the future, that she really wants to get to know new people, and she never said that before./…/. Now she has more of a future, and she wants to make many friends at the new school.”	Have hope for the future	Having a positive outlook on life while facing struggles	Experiencing joy despite depression
Mother of daughter, ID 16: “I have noticed it with NN when they exercise, it has had a good effect/…/meaning physical activity. It’s like the body’s own reward system is activated when you exercise/…/It should be fun.”	Do things that bring joy	Finding pleasure in daily activities	Experiencing joy despite depression
Mother of daughter, ID 21: “It’s both [having energy for] school and leisure activities. So, you can do what you want without being limited by feeling unwell or anything.”	Have energy	Balancing body and mind	Supporting well-being despite depression
Father of daughter, ID 19: “[Health is] about regular sleep, exercise, and food. If you can keep those at a good level, I think you get everything else automatically, or at least most of it.”	Maintain good levels of sleep, exercise, and nutrition	Maintaining healthy habits	Supporting well-being despite depression

## Data Availability

The datasets generated during and/or analyzed during the current study are not publicly available due to legal and ethical restrictions surrounding participant confidentiality. De-identified excerpts of the data can be made available from the corresponding author at a reasonable request. Requests for data access may be subject to approval by the relevant ethics committee. For further information, please contact the corresponding author.

## Supplementary Materials

The following supporting information can be downloaded at: https://www.mdpi.com/article/10.3390/children12091267/s1, COREQ (COnsolidated criteria for REporting Qualitative research) Checklist.
