# Supplementary material for: Parents’ Perceptions of What Health Means for Adolescents with Depression—A Qualitative Study
Source: Children (Basel). 2025 Sep 21;12(9):1267. doi: 10.3390/children12091267 (PMC12468686; doi:10.3390/children12091267)
Supplement: Supplementary file 1 [file children-12-01267-s001.zip › children-3838690-supplementary.pdf]

COREQ (COnsolidated criteria for REporting Qualitative research) Checklist

Title: Parents' perceptions of what health means for adolescents with depression – a qualitative study.

Topic	Item No.	Guide Questions/Description		Reported on Page No.
**Domain 1: Research team and reflexivity**				
Personal characteristics				
Interviewer/facilitator	1	Which author/s conducted the interview or focus group?		Page 4
Credentials	2	What were the researcher's credentials? E.g. PhD, MD		Page 6
Occupation	3	What was their occupation at the time of the study?		Page 6
Gender	4	Was the researcher male or female?		Title page
Experience and training	5	What experience or training did the researcher have?		Page 4
Relationship with participants				
Relationship established	6	Was a relationship established prior to study commencement?		Page 4
Participant knowledge of the interviewer	7	What did the participants know about the researcher? e.g. personal goals, reasons for doing the research		Page 6
Interviewer characteristics	8	What characteristics were reported about the inter viewer/facilitator? e.g. Bias, assumptions, reasons and interests in the research topic		Page 4
**Domain 2: Study design**				
Theoretical framework				
Methodological orientationand Theory	9	What methodological orientation was stated to underpin the study? e.g.grounded theory, discourse analysis, ethnography, phenomenology, content analysis		Page 3
Participant selection				
Sampling	10	How were participants selected? e.g. purposive, convenience, consecutive, snowball		Page 3
Method of approach	11	How were participants approached? e.g. face-to-face, telephone, mail, email		Page 4
Sample size	12	How many participants were in the study?		Page 4
Non-participation	13	How many people refused to participate or dropped out? Reasons?		Page 4
Setting				
Setting of data collection	14	Where was the data collected? e.g. home, clinic, workplace		Page 4
Presence of non-participants	15	Was anyone else present besides the participants and researchers?		No
Description of sample	16	What are the important characteristics of the sample? e.g. demographic data, date		Table 1
Data collection				

Interview guide	17	Were questions, prompts, guides provided by the authors? Was it pilot tested?		Page 4
Repeat interviews	18	Were repeat interviews carried out? If yes, how many?		No
Audio/visual recording	19	Did the research use audio or visual recording to collect the data?		Page 4
Field notes	20	Were field notes made during and/or after the interview or focus group?		No
Duration	21	What was the duration of the interviews or focus group?		Page 4
Data saturation	22	Was data saturation discussed?		Page 4
Transcripts returned	23	Were transcripts returned to participants for comment and/or correction?		No
**Domain 3: analysis and findings**				
Data analysis				
Number of data coders	24	How many data coders coded the data?		Page 4
Description of the coding tree	25	Did authors provide a description of the coding tree?		Table 2
Derivation of themes	26	Were themes identified in advance or derived from the data?		Page 4
Software	27	What software, if applicable, was used to manage the data?		Page 4
Participant checking	28	Did participants provide feedback on the findings?		No
Reporting				
Quotations presented	29	Were participant quotations presented to illustrate the themes/findings? Was each quotation identified? e.g. participant number		Page 6-11
Data and findings consistent	30	Was there consistency between the data presented and the findings?		Yes
Clarity of major themes	31	Were major themes clearly presented in the findings?		Page 6-11
Clarity of minor themes	32	Is there a description of diverse cases or discussion of minor themes?		Page 6-11

Tong A, Sainsbury P, Craig J. Consolidated criteria for reporting qualitative research (COREQ): a 32-item checklist for interviews and focus groups. *International Journal for Quality in Health Care*. 2007. Volume 19, Number 6 pp. 349 - 357
